# Multimodal medical image fusion using convolutional neural network and extreme learning machine

**DOI:** 10.3389/fnbot.2022.1050981

**Published:** 2022-11-16

**Authors:** Weiwei Kong, Chi Li, Yang Lei

**Affiliations:** ^1^School of Computer Science and Technology, Xi'an University of Posts and Telecommunications, Xi'an, China; ^2^Shaanxi Key Laboratory of Network Data Analysis and Intelligent Processing, Xi'an, China; ^3^Xi'an Key Laboratory of Big Data and Intelligent Computing, Xi'an, China; ^4^College of Cryptography Engineering, Engineering University of PAP, Xi'an, China

**Keywords:** image fusion, modality, multimodal medical image, convolutional neural network, extreme learning machine

## Abstract

The emergence of multimodal medical imaging technology greatly increases the accuracy of clinical diagnosis and etiological analysis. Nevertheless, each medical imaging modal unavoidably has its own limitations, so the fusion of multimodal medical images may become an effective solution. In this paper, a novel fusion method on the multimodal medical images exploiting convolutional neural network (CNN) and extreme learning machine (ELM) is proposed. As a typical representative in deep learning, CNN has been gaining more and more popularity in the field of image processing. However, CNN often suffers from several drawbacks, such as high computational costs and intensive human interventions. To this end, the model of convolutional extreme learning machine (CELM) is constructed by incorporating ELM into the traditional CNN model. CELM serves as an important tool to extract and capture the features of the source images from a variety of different angles. The final fused image can be obtained by integrating the significant features together. Experimental results indicate that, the proposed method is not only helpful to enhance the accuracy of the lesion detection and localization, but also superior to the current state-of-the-art ones in terms of both subjective visual performance and objective criteria.

## Introduction

As is well known, the accuracy of lesion detection and localization is crucial during the whole clinical diagnosis and treatment. So far, the rapid growth of medical imaging technologies such as computed tomography (CT), magnetic resonance imaging (MRI), positron emission tomography (PET) and single-photon emission computed tomography (SPECT) has provided us much richer information on the physical condition. CT can accurately detect the slight differences of the bone density in a transection plane, which is regarded as an ideal way to observe the lesions of the bone. Nevertheless, its capacity of the tissue characterization is weak. The information of the soft tissue can be better visualized in MRI images, but the movement information such as the body metabolism cannot be found. Unlike MRI, PET images can reflect the activity of the life metabolism through the accumulation of certain substance so as to achieve the purpose of diagnosis, but they are often with lower resolution. The main advantage of SPECT is to demonstrate the changes in blood flow, function and the metabolism of organs or diseases, which is beneficial to the early and specific diagnosis of the disease. Obviously, due to the respective different mechanism, each imaging modality unavoidably has its characteristics and inherent drawbacks. To this end, the fusion of the medical images with multiple different modalities may be an effective solution, because it can not only combine the advantages together to accurately implement the localization and description of the lesion, but also reduce the storage cost of the patient information database.

Recently, a variety of fusion methods on multimodal medical images have been proposed during the past decades. Basically, these methods can be mainly grouped into the following categories, namely spatial domain-based methods, transform domain-based methods, soft computing-based methods, and deep learning-based ones.

The representative spatial domain-based methods include simple averaging, maximum choosing, principal component analysis (PCA) (He et al., 2010) and so on. Although most of the above methods have comparatively high operating speed and simple framework, they often tend to suffer from contrast reduction and spectrum distortion in the final fused image. Therefore, the pure spatial domain-based methods are rarely used at present.

Unlike spatial domain-based methods, the core scheme of transform domain-based methods usually consists of three steps. Firstly, the source image is converted to the frequency domain to get several sub-images which commonly contain one approximation image with low-pass coefficients and several detail images with high-pass coefficients. Secondly, certain rules are adopted to complete the fusion of sub-images at corresponding stages. Finally, the final fused image is reconstructed. The classical methods include, but are not limited to, Laplacian pyramid transform, discrete wavelet transform (DWT), contourlet transform, shearlet transform and so on, which have pioneered the use of transform domain-based concept. However, with further in-depth research on the medical image fusion, the defects of the above classical methods are gradually revealed. Under this background, a series of improved versions have been presented in the past decade. Du et al. (2016) introduced union Laplacian pyramid to complete the fusion of medical images. Some improved versions of DWT such as dual tree complex wavelet transform (DT-CWT) (Yu et al., 2016), non-subsampled rotated complex wavelet transform (NSRCxWT) (Chavan et al., 2017), discrete stationary wavelet transform (DSWT) (Ganasala and Prasad, 2020a; Chao et al., 2022) were presented to complete the fusion of medical images. Compared with DWT, these three new versions share both the redundancy feature and the shift-invariance property, which effectively avoid the Gibbs phenomenon in DWT. Similarly, in order to overcome the absence of shift-invariance in the original contourlet transform and shearlet transform, the corresponding improved versions namely non-subsampled contourlet transform (NSCT) and non-subsampled shearlet transform (NSST) were proposed successively. In comparison to the aforementioned transform domain-based methods, NSCT and NSST have both manifested competitive fusion performance due to their flexible structures. Zhu et al. (2019) combined NSCT, phase congruency and local Laplacian energy together to present a novel fusion method for multi-modality medical images. Liu X. et al. (2017), Liu et al. (2018) proposed two NSST-based methods to fuse the CT and MRI images.

In addition to spatial domain-based methods and transform domain-based methods, extensive work has also been conducted with soft computing-based methods dedicated to multimodal medical image fusion. A great many representative models, including dictionary learning model (Zhu et al., 2016; Li et al., 2018), gray wolf optimization (Daniel, 2018), fuzzy theory (Yang et al., 2019), pulse coupled neural network (Liu X. et al., 2016; Xu et al., 2016), sparse representation (Liu and Wang, 2015; Liu Y. et al., 2016), total variation (Zhao and Lu, 2017), guided filter (Li et al., 2019; Zhang et al., 2021), genetic algorithm (Kavitha and Thyagharajan, 2017; Arif and Wang, 2020), compressed sensing (Ding et al., 2019), structure tensor (Du et al., 2020c), local extrema (Du et al., 2020b), Otsu's method (Du et al., 2020a) and so on, were successfully used to fuse the medical images.

Since the transform domain-based methods and soft computing-based methods have both manifested to be promising in the field of medical image fusion, some novel hybrid methods have also been presented in recent years. Jiang et al. (2018) combined interval type-2 fuzzy sets with NSST to complete the fusion task of multi-sensor images. Gao et al. (2021) proposed a fusion method based on particle swarm optimization optimized fuzzy logic in NSST domain. Asha et al. (2019) constructed a novel fusion scheme based on NSST and gray wolf optimization. Singh and Anand (2020) employed NSST to decompose the source images, and then used sparse representation and dictionary learning model to fuse the sub-images. Yin et al. (2019) and Zhang et al. (2020) each proposed a NSST-PCNN based fusion method for medical images. The guided filter was combined with NSST to deal with the issue of multi sensor image fusion (Ganasala and Prasad, 2020b). Zhu et al. (2022) combined the advantages of both spatial domain and transform domain methods to construct an efficient hybrid image fusion method. Besides, the collective view of the applicability and progress of information fusion techniques in medical imaging were reviewed respectively in Hermessi et al. (2021) and Azam et al. (2022).

In recent years, the deep learning-based methods play significant roles in the field of medical image fusion, and have been gaining more and more popularity in both the academic and industry community. In 2017, convolutional neural network (CNN) was firstly introduced into the area of image fusion by Liu Y. et al. (2017). Fan et al. (2019) deeply researched the semantic information of the medical image with different modalities, and proposed a semantic-based fusion method for medical images. Aside from CNN, another representative deep learning model namely generative adversarial network (GAN) was used to deal with the issue of image fusion in 2019 (Ma et al., 2019). The unsupervised deep networks for medical image fusion were presented in references (Jung et al., 2020; Fu et al., 2021; Xu and Ma, 2021; Shi et al., 2022). Goyal et al. (2022) combined transform domain-based methods and deep learning-based methods together to present a composite method for image fusion and denoising.

After consulting a great deal of literature, we found that how much information from the original source medical images is retained in the final fused image greatly determines the image quality, which is crucial to the further clinical diagnosis and treatment. So far, the single transformed domain-based methods and relevant hybrid ones have been widely employed to deal with the fusion issue of medical images. However, the transformed domain-based methods may introduce the frequency distortion into the fused image. With the rapid development of the deep learning theory and its reasonable biological background, more and more attention is being paid to the deep learning-based methods such as CNN. Therefore, we desire to develop a novel fusion method based on CNN to fuse the medical images. It is noteworthy that each single theory always has its advantages and disadvantages and deep learning is no exception, which is usually accompanied by a huge amount of computational costs. To this end, we need to construct or adopt some model to reduce the computational complexity as much as possible.

In this paper, a novel fusion method on the multimodal medical images exploiting CNN and extreme learning machine (ELM) (Huang et al., 2006, 2012; Feng et al., 2009) is proposed. On the one hand, since the nature of the medical image fusion can be regarded as the classification problem, the existing successful experiences of CNN can be fully applied. On the other hand, due to a great many parameters, the computational cost of CNN is high. ELM is a single hidden layer feed-forward network, and its algorithm complexity is very low. Besides, since ELM belongs to a convex optimization problem, it will not fall into the local optimum. Therefore, ELM is utilized to improve the traditional CNN model in this paper.

The main contributions of this paper can be summarized as follows.

- A novel method based on CNN and ELM is proposed to deal with the fusion issue of multimodal medical images.
- It is proved that, apart from the area of multi-focus image fusion, the CNN model can also be used in the field of multimodal medical image fusion.
- The traditional CNN model is integrated with ELM to be a modified version called convolutional extreme learning machine (CELM) which has not only much better performance, but also much faster running speed.
- Experimental results demonstrate that the proposed method has obvious superiorities over the current typical ones in terms of both gray image fusion and color image fusion, which is beneficial to obviously enhancing the precision of disease detection and diagnosis directly.

The rest of this paper is organized as follows. The involved theories of CNN and ELM are reviewed in Related work section followed by the proposed multimodal medical image fusion framework in Proposed method section. Experimental results with relevant analysis are reported in fourth section. In Conclusions section, the concluding remarks are given in the end.

## Related work

The models relevant to the proposed method are introduced in this section. The two important concepts, namely CNN and ELM are briefly reviewed as follows.

### Convolutional neural network

**Figure 1:**
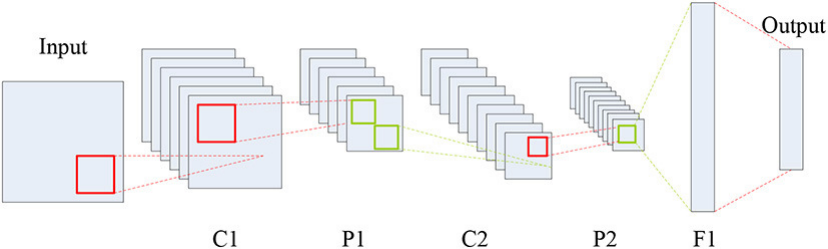
Typical CNN structure.

As a representative neural network in the field of deep learning, CNN aims to learn a multistage feature representation of the input data, and each stage usually consists of a series of feature maps connected *via* different types of calculations such as convolution, pooling and full connection. As shown in Figure 1, a typical CNN structure is composed of five types of components including the input layer, convolution layers, pooling layers, full connection layer, and the output layer.

In Figure 1, C, P and F denote the convolution, max-pooling and full connection operations, respectively, which can generate a series of feature maps. Each coefficient in the feature maps is known as a neuron. Clearly, CNN is an end-to-end system. The roles of the three types of layers, namely convolution, pooling and full connection, can be summarized as feature extraction, feature selection, and the classifier.

Here, the input data is a two-dimensional image. The neurons between the adjacent stages are connected by the operations of convolution and pooling, so that the number of the parameters to be learned declines a lot. The mathematical expression of the convolution layer can be described as:


(1) $$\begin{array}{l} y^{j}=b^{j}+\underset{i}{\sum }k^{ij}\;*\;x^{i} \end{array}$$

where *k*^*ij*^ and *b*^*j*^ are the convolution kernel and the bias, respectively. The symbol ^*^ denotes the 2D convolution. *x*^*i*^ is the *i*th input feature map and *y*^*j*^ is the *j*th output one.

In fact, during the convolution course, the non-linear activation is also conducted. The common activation functions include sigmoid function, rectified linear units (ReLU), and so on. Here, ReLU is adopted whose mathematical expression can be written as:


(2) $$\begin{array}{l} y^{j}=max\left(0,b^{j}+\underset{i}{\sum }k^{ij}\;*\;x^{i}\right) \end{array}$$

In CNN, the convolution layer is usually followed by the pooling layer. The common pooling rules include max-pooling and average-pooling, which can select the maximum or the average value of a certain region to form new feature maps. Due to the special mechanism of the pooling layer, it can bring some desirable invariance such as translation and rotation. Moreover, it can also decrease the dimension of the feature maps which is favorable for reducing the computational costs as well as the memory consumption.

Through the alternation of multiple convolution and pooling layers, CNN relies on the full connection layer to classify the extracted features to obtain the probability distribution *Y* based on the input. In fact, CNN can be viewed as a converter where the original matrix *X* can be mapped into a new feature expression *Y* after multiple stages of data transformation and dimension reduction. The mathematical expression can be written as:


(3) $$Y(i)\text{ = }P\text{(}L\text{ =}l_{i}\text{|}H_{0}\text{; (}k\text{,}b\text{))}$$

where *H*_0_ is the original matrix, and the training objective of CNN is to minimize the loss function *L*(*k, b*). *k* and *b* are the convolution kernel and the bias, respectively, which can be updated layer by layer *via* the following equations.


(4) $$\begin{array}{l} k_{i}=k_{i}-\eta \frac{\partial E\left(k,b\right)}{\partial k_{i}} \end{array}$$

(5) $$\begin{array}{l} b_{i}=b_{i}-\eta \frac{\partial E\left(k,b\right)}{\partial b_{i}} \end{array}$$

(6) $$\begin{array}{l} E\left(k,b\right)=L\left(k,b\right)+\frac{\lambda }{2}k^{T}k \end{array}$$

where λ and η denote the weight decay parameter and the learning rate, respectively.

According to the mechanism of CNN mentioned above, the important features of the image can be classified. Some fused methods for multi-focus images based on CNN have been published in recent years. Although CNN-based fusion methods have been gaining more and more popularity, their inherent problems such as being prone to local minima, intensive manual intervention and the waste of the computing resources still cannot be ignored.

### Extreme learning machine

Different from the conventional neural networks, ELM is a single hidden layer feed-forward neural network. It is generally known that most current neural networks have many knotty drawbacks. (a) The training speed is slow. (b) It is easy for them to be trapped into the local optimum. (c) The learning rate is very sensitive to the parameters selection. Fortunately, ELM is able to generate randomly the weights between the input and the hidden layer as well as the threshold of the neuron in the hidden layer, and the weights adjustment is totally unnecessary. In other words, the optimum solution can be obtained, provided the neuron number in the hidden layer is given.

Suppose *N* training samples (**x**_***i***_, **t**_***i***_) and a single layer feed-forward neural network with *L* neurons in the hidden layers and *M* ones in the output layers. The concrete steps of the learning *via* ELM are as follows.

Step 1: The node parameters are allocated randomly, which is independent of the input data.
Step 2: Computing the output matrix **h**(***x***) = [*g*_1_(***x***), …, *g*_*L*_(***x***)]^*T*^ of the hidden layers for ***x***. Obviously, the size of **h**(***x***) is *N*×*M*, which is the mapping result from *N* input data to *L* neurons in essence.
Step 3: Computing the output weights matrix **β** = [**β**_1_, …, **β**_*L*_]^*T*^. **β**=**H**^*T*^**T**. **H** = [**h**^*T*^(**x**_1_), …, **h**^*T*^(***x***_*N*_)]^*T*^, and **T** = [**t**_1_, **t**_2_, …, **t**_*N*_]^*T*^ is the training objective. The output weights matrix **β** can be obtained by using the regularized least squares method as follows.
  (7) $$\begin{array}{l} \beta ={\left(\frac{I}{C}+H^{T}H\right)}^{-1}H^{T}T \end{array}$$
  where *C* is the regularization coefficient.

**Figure 2:**
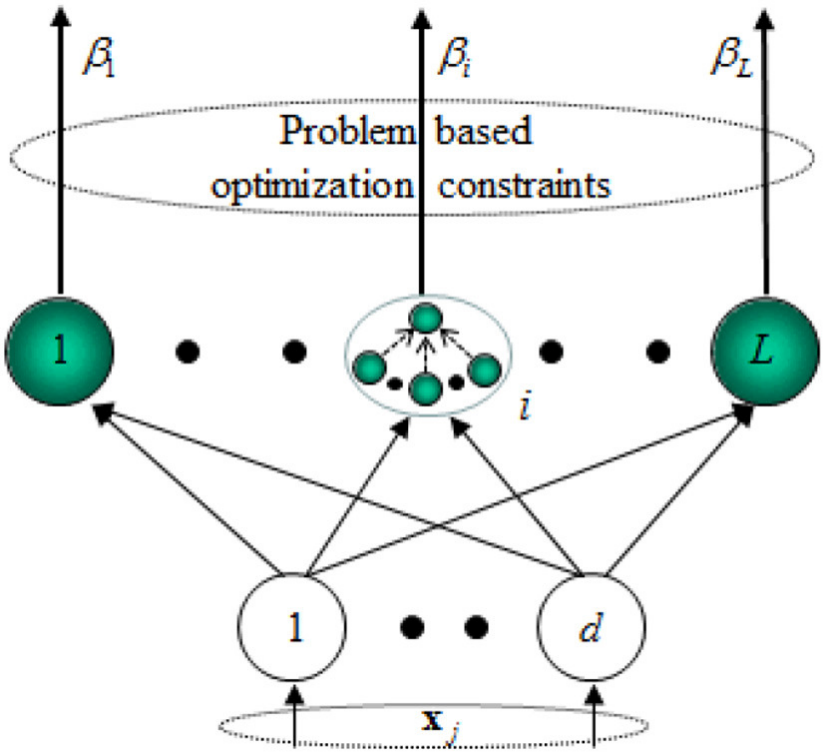
Scheme of the ELM feature mapping.

Besides, a hidden neuron of ELM can be a sub-network of several neurons. The scheme of the ELM feature mapping is shown in Figure 2.

## Proposed method

In this section, the proposed fusion method for multimodal medical images based on CNN and ELM is presented in detail. The concrete content can be divided into three subsections, including the structure of convolutional extreme learning machine (CELM), network design, and the fusion schemes.

### Structure of CELM

As described in Related work section, we can reach several conclusions as follows.

- It is feasible to utilize CNN to deal with the issue of image fusion.
- There are still inherent drawbacks in the traditional CNN model, so it has large development potentiality.
- ELM not only owns many superiorities over other current neural networks, but also shares great similarities with CNN in structure.

**Figure 3:**
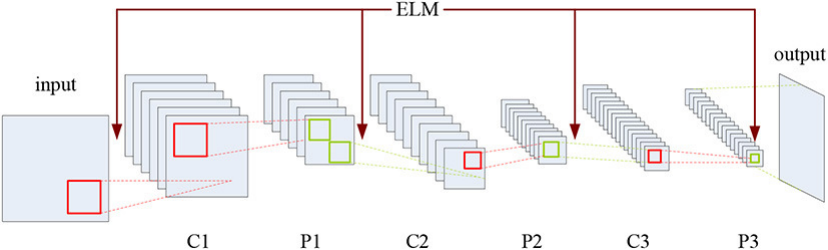
Structure of CELM.

Therefore, it is sensible to integrate CNN with ELM to combine the both advantages together, which may also introduce a novel and more effective solution to the fusion of multimodal medical images. To this end, the CELM model is proposed in this paper, whose structure is shown in Figure 3.

As shown in Figure 3, C and P denote the convolution and pooling operations, respectively, and the mechanism of ELM has been added into the CNN structure. CELM is composed of an input layer, an output layer, and several hidden layers where the convolution layers and the pooling layers alternately appear. The convolution layer consists of several maps recording the features of the previous layer *via* several different convolution kernels. The pooling layer introduces the translation invariance into the network, and the dimension of the feature map in the previous layer will also decrease. Meanwhile, the number of the feature maps in the pooling layer always equals to the one in the previous convolution layer. It is noteworthy that, except for the first convolution layer, the neurons of the feature map in the convolution layer are all connected to all the feature maps in the previous pooling layer, while the ones in the pooling layer are only connected to the corresponding feature maps in the previous convolution layer. As for the original full connection layer in the original CNN model, it has been replaced by the global average pooling layer (Lin et al., 2014), which is favorable for sharply cutting down the number of parameters.

With regard to the feature extraction, ELM can randomly generate the weights between the input layer and the first convolution layer as well as the ones between the pooling layer and the following convolution layer, as shown in Figure 3. Here, we suppose that there are two original multimodal medical images denoted by *A* and *B*, respectively. If the source images are color ones, we can convert them into gray ones or deal with them in different color spaces, which will be involved in a later section.

In CELM, the weights are viewed to be agreeing with the normal distribution, and the weight matrix can be obtained as follows.


(8) $$\begin{array}{l} P=\left[{\hat{p}}^{1},{\hat{p}}^{2},\ldots ,{\hat{p}}^{i},\ldots {\hat{p}}^{N}\right],1\leq i\leq N \end{array}$$

where **P** is the initial weight matrix, *N* is the number of convolution kernels, and the size of each element in Equation (8) is *r* × *r*. Therefore, if the size of the previous layer is *k* × *k*, the size of the corresponding feature map would be (*k* – *r* + 1) × (*k* – *r* + 1).

The convolution node on the point at (*x, y*) on the *i*th feature map can be obtained as


(9) $$\begin{array}{l} c_{x,y,i}\left(\Theta \right)=\sum_{m=1}^{r}\sum_{n=1}^{r}\Theta_{x+m-1,y+n-1}\cdot p_{m,n}^{i} \end{array}$$

where “Θ” denotes the source image *A* or *B*.

As for the pooling layer, the max-pooling strategy is adopted except the last layer. The pooling node on the point at (*u, v*) on the *j*th pooling map can be obtained as:


(10) $$\begin{array}{l} c_{u,v,j}\left(\Theta \right)=max\left[c_{x,y,i}\right],x,y=u-z,\ldots ,u+z \end{array}$$

where *z* denotes the pooling size.

**Figure 4:**
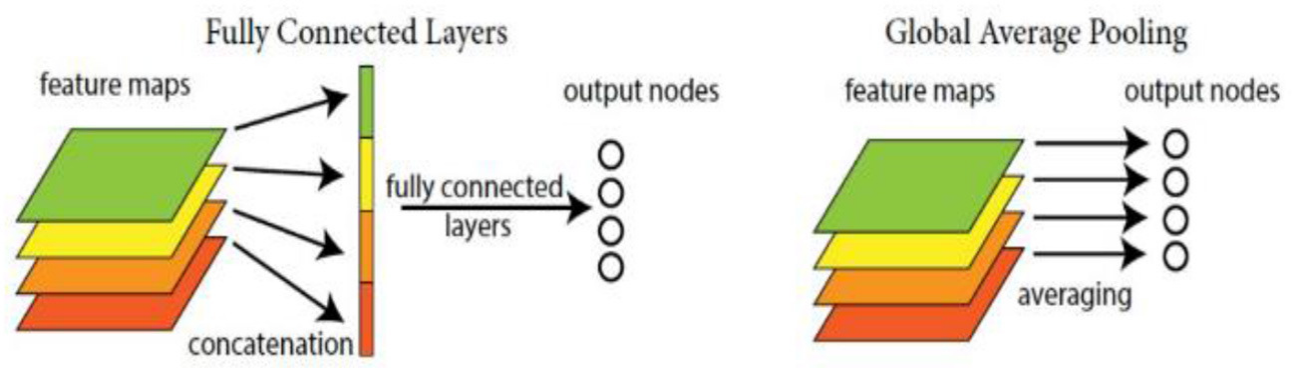
Diagram of the global average pooling layer.

Due to involving a large number of parameters, the original full connection layer in CNN is substituted for the global average pooling one here, so that we can directly treat the feature maps as the category confidence ones, and save the computational costs and storage space. The diagram of the global average pooling layer is shown in Figure 4.

### Network design

In this work, multimodal medical image fusion is regarded as a classification problem. CELM is able to provide the output ranging from 0 to 1 according to a series of image patches {p_*A*_, p_*B*_}. As is known, the essence of image fusion is to extract the important information from the source images and then fuse it into a single one. Fortunately, CELM can just lead us to find the representative information *via* classification. Specifically, the output near to 1 indicates the information in p_*A*_ has better reference value, while the information in p_*B*_ seems more typical if the output is close to 0. Therefore, the pair of the patches {p_*A*_, p_*B*_} from the same scene can be used as the training samples in CELM. For example, if the information in p_*A*_ is more valuable than that in p_*B*_, the corresponding label is set to 1, otherwise the label is set to 0. For sake of maintaining the image information integrity, the whole source medical images are input into the CELM as a whole rather than dividing them into a series of patches. The results in the output layer can provide the scores reflecting the information importance in the source images.

As for the details of the network, two important points need to be made. (a) The network framework can be mainly categorized into three types according to the reference (Zagoruyko and Komodakis, 2015), namely siamese, pseudo-siamese and two-channel. The last type just has a trunk rather than branches. The difference between siamese and pseudo-siamese lies in whether the weights of the branches of them are the same or not. Here, the siamese type is chosen as the network framework in this paper, the reason for which can be summarized as follows. Firstly, due to the weight sharing, the network training course is easy and timesaving. Secondly, take the fusion course of two source images for example, two branches with the same weights indicate the same schemes of feature extracting are used for these two images, which is just consistent with the process of image fusion. (b) The final fusion performance has something to do with the size of the input patch. For example, when the patch size is set to 64 × 64, the classification ability of the network is relatively high since much more image information is taken into consideration. According to Farfade et al. (2015), there is the 2-power law relation between the kernel stride and the number of the max-pooling layer. In other words, if there are four max-pooling layers, the corresponding stride is 2^4^ = 16 pixels. Obviously, the final fused image will suffer from blocky effects. Therefore, in order to guarantee the classification ability and remove the blocky effects as much as possible, the patch size is set to 32 × 32 in this paper.

**Figure 5:**
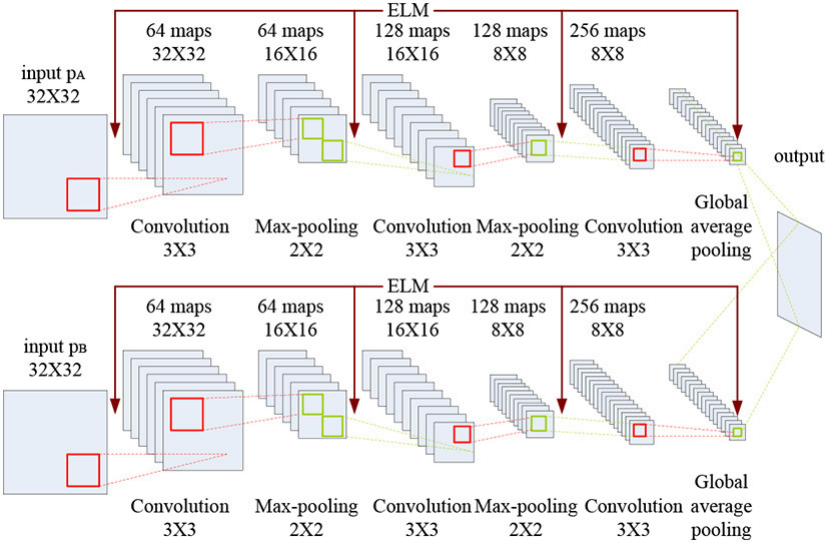
CELM diagram used for multimodal medical image fusion.

The CELM diagram used for multimodal medical image fusion is shown in Figure 5.

As indicated in Figure 5, each branch consists of three convolution layers, two max-pooling layers and a global average pooling layer. The kernel size and the stride of the convolution layer are set to 3 × 3 and 1, while the corresponding values of the max-pooling layer are set to 2 × 2 and 2. Here, the global average pooling is used for realizing the function of the original full connection layer in CNN, and the 256 feature maps are obtained for classification.

### Fusion schemes

In this paper, the training datasets of CELM are from the website www.ctisus.com, which is the premier radiological website dedicated to multimodal scanning. This website has an incredible library of content ranging from multimodal scan protocols, lectures, case studies, medical illustrations, and a monthly quiz. CTisus.com provides the latest in radiology technology and 3D imaging information, and uploads new content daily.

After constructing the CELM, the fusion issue of the multimodal medical images can be achieved. The specific implementation process consists of two stages, namely 1-stage and 2-stage. Here, we only take the fusion of two images into consideration, and the method can be extended to the case of the fusion of more than two images.

During the 1-stage, the concrete steps are as follows.

**Input:** Patches of the multimodal medical images to be fused.

**Output:** The 1-stage fused image.

**Initialization:** The CELM depicted in Figure 5.

**Step 1.1:** The patch of 32 × 32 pixels are fed into the CELM.

**Step 1.2:** By using the two convolution layers, we can obtain 64 and 128 feature maps, respectively. The kernel sizes of the two convolution layers are set to 3 × 3, and the strides of the convolution layers are set to 1.

**Step 1.3:** The kernel sizes of the two max-pooling layers are both set to 2 × 2, and the strides of the convolution layers are set to 2. And 128 feature maps can be obtained.

**Step 1.4:** The 128 feature maps are fed into another third convolution layer with the size of 3 × 3 to generate 256 feature maps.

**Step 1.5:** The global average pooling layer is used to deal with the 256 feature maps in Step 1.4.

**Step 1.6:** Guarantee that all the pixels of the source images are performed by CELM, and the output can be obtained as:


(11) $$label(i,j)=\{\begin{array}{c} 1,\;if\;A\left(i,j\right)is\;better\;than\;B\left(i,j\right)\\ 0,\;otherwise \end{array}$$

(12) $$F(i,j)=\{\begin{array}{c} A\left(i,j\right),\;if\;label\left(i,j\right)=1\\ B\left(i,j\right),\;if\;label\left(i,j\right)\neq 1 \end{array}$$

where “*label*” is the classification result of CELM. *A, B* and *F* denote the two source images and the final fused one, respectively. (*i, j*) is the coordinate of the pixel in the image.

It should be noted that there will be inconsistency during the fused image, namely a pixel from the source image *A* may be surrounded by a great many counterparts from *B*.

In order to overcome the problem mentioned above, a consistency matrix denoted by *C* is defined here to describe the ownership of the pixels. If the pixel *F*(*i, j*) is from *A*, the value of the corresponding element *C*(*i, j*) is set to 1, otherwise the value is 0. Then, a filter whose size and stride are 3 × 3 and 1 respectively is used. In the 3 × 3 window, three cases may appear. (a) If the sum of the surrounding eight elements in *C* is greater than or equal to five, the corresponding pixel in *A* will be selected as the counterpart in *F*. (b) If the sum of the surrounding eight elements in *C* is less than or equal to three, the corresponding pixel in *B* will be selected as the counterpart in *F*. (c) If the sum of the surrounding eight elements in *C* is four, the original value in *F* will remain unchanged.

**Figure 6:**
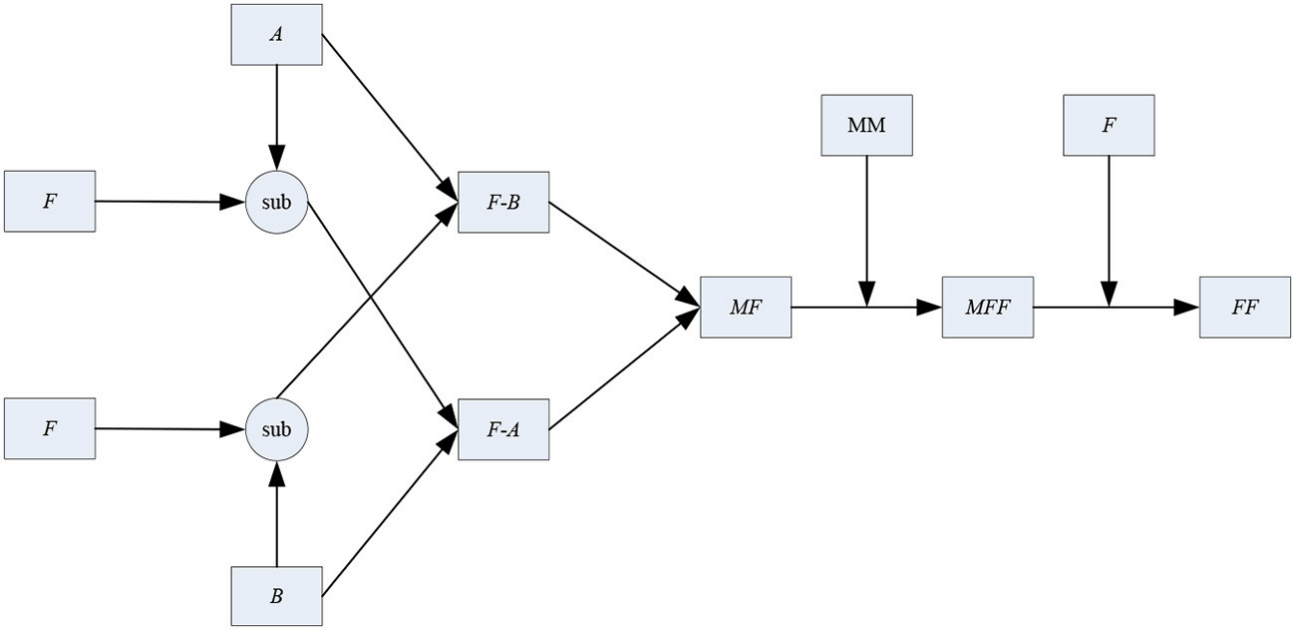
Diagram of the two-stage.

After the 1-stage, the initial fused image can be obtained. However, unlike the fusion of other types of images, higher requirements and standards are needed in the fusion course of multimodal medical images to enhance the precision of lesion detection and diagnosis. In the 2-stage, the connection between the two source images and the initial fused one is analyzed and discussed further. The diagram of the 2-stage is shown in Figure 6.

As shown in Figure 6, *A, B, F* and *FF* denote the two source images, the initial fused one and the final fused one, respectively. “sub” is the subtraction operator. “*F*-*A*” stands for the subtraction result between *F* and *A*. Similarly, “*F*-*B*” stands for the subtraction result between *F* and *B*. *MF* and *MFF* denote the binary mapping of the images *F* and *FF*. MM is the abbreviation of mathematical morphology.

In this paper, the simple subtraction operator is used to measure the similarity between the initial fused image and the source one. The concrete steps of the 2-stage are as follows.

**Input:** Two source images denoted by *A* and *B*, and the initial fused image *F*.

**Output:** The 2-stage fusion result *FF*.

**Initialization:** The two source images and the initial fused one are given.

**Step 2.1:** The subtraction operation is conducted between *A* and *F* to generate the image *F*-*A*. Similarly, the image *F*-*B* can be also obtained.

“*F*-*A*” and “*F*-*B*” can describe the extent of feature extracting from the other original source image.

**Step 2.2:** Compute the value of root mean square error (RMSE) between “*F*-*A*” and “*B*” to obtain *RMSE*__*F*_−A, *B*_. Meanwhile, *RMSE*__*F*_−B, *A*_ can also be computed. Here, the size of the window used to compute RMSE is 5 × 5.

**Step 2.3:** Construct a new matrix *MF* with the same size as *F*. The elements of *MF* can be determined as:


(13) $$MF(i,j)=\{\begin{array}{c} 1,\;if\;RMSE_{F-B,A}>RMSE_{F-A,B}\\ 0,\;otherwise \end{array}$$

where *MF*(*i, j*) = 1 indicates that the difference between *F*-*B* and *A* is more obvious than that between *F*-*A* and *B*. In other words, more information should be fused in *A* than that in *B*, otherwise we may should place more emphasis on *B* rather than *A*.

**Step 2.4:** With the help of MM, *MF* is optimized by a series of opening and closing operators to smooth over the object outlines and the connection between each other. Here, the structure element is a square identity matrix of the size 5 × 5. The modified mapping denoted by *MFF* can be obtained.

**Step 2.5:** *MFF* and *F* are both taken into account to determine the final fused image *FF*. Please note that compared with the requirements in the 1-stage, the modification condition is more rigorous here. The reason for it lies in that the initial fused image have been already obtained in the 1-stage, while the main objective of the 2-stage aims to further optimization. The elements of *FF* can be optimized as:


(14) $$FF(i,j)=\{\begin{array}{c} 1,\;if\;MFF\left(i,j\right)=1\;and\;sum\left(i,j\right)=8\\ 0,\;if\;MFF\left(i,j\right)=0\;and\;sum\left(i,j\right)=0\\ F(i,j),\;otherwise \end{array}$$

where “sum” denotes the sum of the elements surrounding (*i, j*) in *MFF*. The window is of size 3 × 3. As Equation (14), if and only if the elements in the window are all from the same source image, the corresponding value in the initial fused image may be modified. Otherwise, the element will still remain unchanged.

It is also noteworthy that if the source images are color ones, we need to convert them into gray ones or deal with them in different color spaces. The color is usually characterized by three independent attributes, which interact on each other to form a spatial coordinate called color space. The color space can be divided into two categories including primary color space, and color brightness separation color space according to the basic structure. RGB and YUV are the typical representatives of the above categories respectively.

RGB mode is an additive one with luminescent screen, while CMYK mode is a printing subtractive one with reflective color. IHS mode suffers from spectral information distortion, which easily leads to medical accidents. Unlike the above three common modes, YUV mode can deal with brightness or color without mutual influences, so it depends on neither light nor pigment. Moreover, YUV includes all color modes the human can see in theory, and it is able to make up for the drawbacks of RGB, CMYK and IHS. Therefore, YUV mode is chosen as the color space in this paper.

During the fusion course of medical source images, we may encounter color images, such as SPECT-TI and SPECT-Tc based ones. Under the circumstances, the RGB source image is converted into the YUV version first. Three components including Y, U and V can be obtained. The Y channel describes the brightness information of the image whereas the other two channels cover the color information. The Y component is fused using the proposed scheme followed by the conversion from YUV to RGB to get the final fused image *F*.

## Experimental results with relevant analysis

In order to verify the effectiveness and the superiorities of the proposed method, a series of simulation experiments are performed. Concretely, the section is composed of six parts. The information on the source images to be fused, the methods which are used to be compared with the proposed one, and the experiment settings are given in detail in Experimental setups section. Objective evaluation metrics section lists the objective quantity metrics used in the following experiments. In Experiments on gray and color source images section, the comparisons on the gray images and color ones are conducted in terms of both subjective visual performance and objective quantity results. As the extensive research, the application of the proposed method in other types of source images is also investigated in Applications of the proposed method in other types of source images section followed by the average running time of the proposed method in Average running time of the proposed method section. In the end, the discussions on the potential research directions of the proposed method are given in Discussions on the potential research directions of the proposed method section.

### Experimental setups

**Figure 7:**
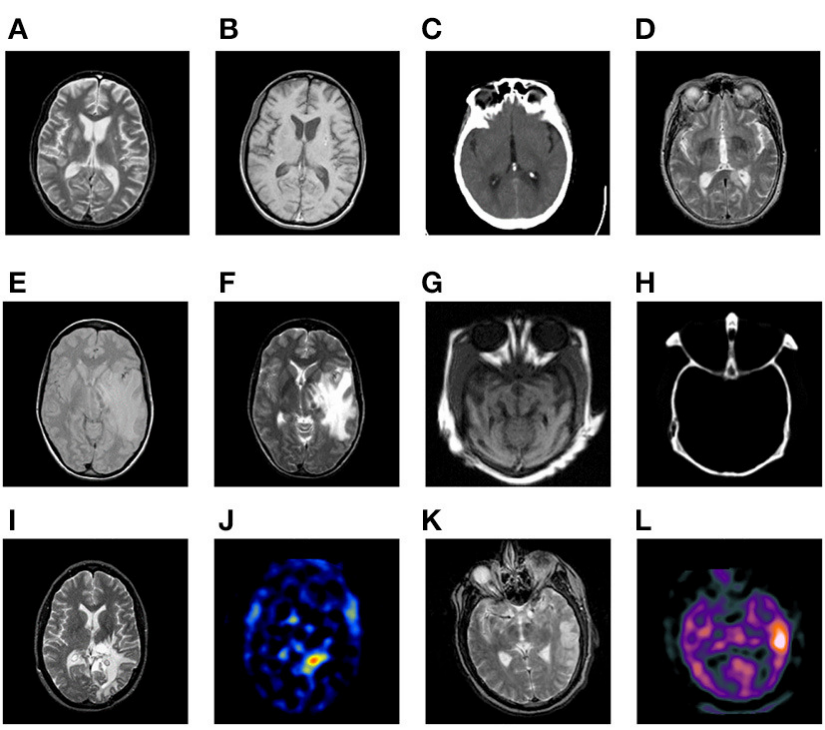
Six pairs of multimodal medical source images. Pair I **(A,B)**. Pair II **(C,D)**. Pair III **(E,F)**. Pair IV **(G,H)**. Pair V **(I,J)**. Pair VI **(K,L)**.

Six pairs of multimodal medical images are used in the following experiments, which are shown in Figure 7. There are several points requiring to be noted. (a) For simplicity, the corresponding pairs of source images are named as Pair I–VI. (b) All the images share the same size of 256 × 256 pixels, and can be downloaded from the Harvard university site^1^ or the Netherland TNO site^2^ (c) From the color perspective, the images in pair I–IV are gray ones covering 256-level gray scale, while the images in pair V–VI such as SPECT ones are in pseudo-color. (d) The images with different modalities own a great deal of complementary information, which is beneficial for increasing the accuracy of the lesion detection and localization.

The proposed method is compared with seven representative and recently published ones, which are the adaptive sparse representation (ASR)-based (Liu and Wang, 2015) one, the convolutional sparse representation (CSR)-based one (Liu Y. et al., 2016), the non-subsampled rotated complex wavelet transform (NSRCxWT)-based one (Chavan et al., 2017), the guided filtering fusion (GFF)-based one (Li et al., 2013), the cross bilateral filter (CBF)-based one (Kumar, 2015), CNN-based one (Liu Y. et al., 2017) and gradient transfer and total variation (GTTV)-based one (Ma et al., 2016). Generally speaking, ASR, CSR and NSRCxWT belong to the scope of TDB, while the other four methods are SCB ones. In order to guarantee the objectivity during the whole process of simulation experiments, the free parameters of the seven methods used to be compared are all set as the original references reported.

### Objective evaluation metrics

As is well known, it is one-sided for us to evaluate the fusion performance only by subjective inspection. The objective quantity evaluation also plays a significant part during the whole process of image fusion. In Liu et al. (2012), the 12 metrics which are recently proposed and typical are fully analyzed and discussed. On the whole, they can be categorized as four types, namely information theory-based metrics, image feature-based metrics, image structural similarity-based metrics, and human perception inspired fusion metrics. In this paper, four metrics each of which is from four different types above respectively are selected to perform the objective evaluation on the final fused results, including spatial frequency (*Q*_*SF*_) (Zheng et al., 2007), Piella's metric (*Q*_*Piella*_) (Piella and Heijmans, 2003), mutual information (*Q*_*MI*_) (Hossny et al., 2008), and Chen-Varshney metric (*Q*_*CV*_) (Chen and Varshney, 2007).

### Experiments on gray and color source images

From the modality perspective, the source images are of six different combinations as follows.

- Pair I (MR-T2 and MR-T1)
- Pair II (CT and MR-T2)
- Pair III (MR-PD and MR-T2)
- Pair IV (CT and MR)
- Pair V (MR-T2 and SPECT-TI)
- Pair VI (MR-T2 and SPECT-Tc)

**Figure 8:**
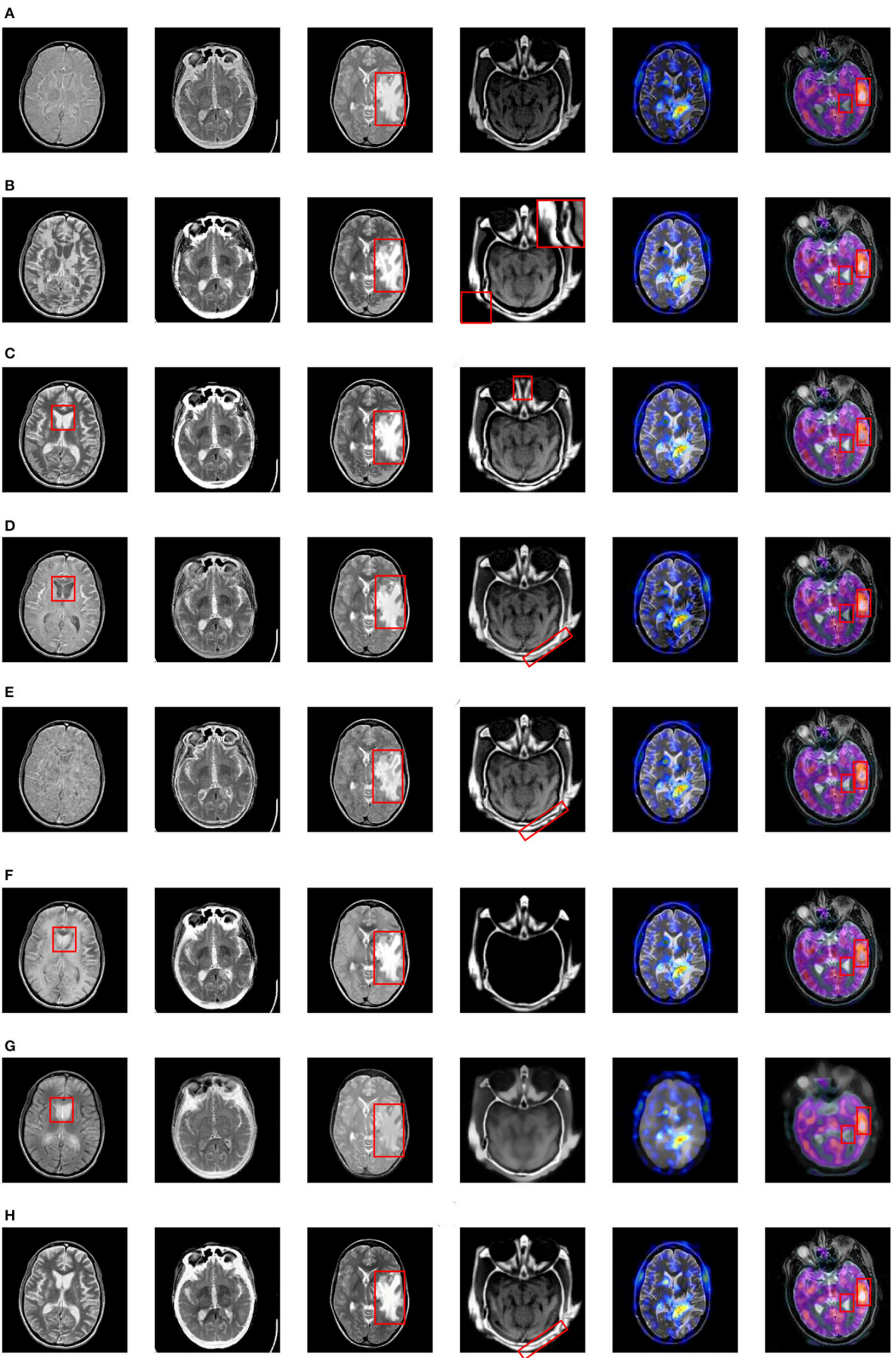
Fusion results based on eight different methods. **(A)** ASR, **(B)** CSR, **(C)** NSRCxWT, **(D)** GFF, **(E)** CBF, **(F)** CNN, **(G)** GTTV, **(H)** Proposed.

The fusion results based on the eight different methods are shown in Figure 8.

As for the fused results on Pair I, the ASR-based and CBF-based methods suffers from poor contrast. A great deal of artifacts can be easily found in the fused image based on CSR. Besides, the information of the source images doesn't obtain a fully expression in the fused images based on GFF, CNN and GTTV (please see the red rectangles), which is very unfavorable to the lesion detection and localization. In comparison, the fused images based on NSRCxWT and the proposed one have much better visual performance. In Pair II, a striking comparison can be easily observed that the outline information in Figure 7C is not adequately described by the other seven methods except the proposed one. In other words, the bright white outline is supposed to appear continuously and obviously in the fused image. As to Pair III, the center-right region can be used as a reference (see the red rectangles). The fused images based on ASR, GFF and GTTV have a relatively low contrast level. What is worse, some artifacts even appear in the fused results based on CSR and CBF. Compared with the above five methods, NSRCxWT, CNN and the proposed one all have satisfactory visual performance. However, through careful observation, it can be found that the proposed method has more superiorities over other two ones in terms of the image texture and the information representation. In Pair IV, the original information of the source CT image is almost lost in the fused images based on ASR, CNN and GTTV. In the fused image based on NSRCxWT, there is also an obvious lack of the source MRI information (see the red rectangles). Similarly, the information locating at the bottom right corner in the CBF-based result is also missing. A terrible indented edge can be noticed in the fused result based on CSR (see the magnified region in the upper right corner). Compared with the other six methods, GFF and the proposed method have much better visual performance, but the latter owns much clearer contours than the former, which can be found in the red rectangles. The experiments on Pair V and Pair VI involve the fusion between the gray image and the color one, and their fused results are also in color. Compared with the gray counterparts, color images are able to offer much more information with no doubt. Pair V describes the case of anaplastic astrocytoma. The significant lesion regions obtain better descriptions in the fused image based on the proposed method than other ones. Pair VI addresses another case. Here, for sake of distinguishing the differences among the eight methods, two regions are selected as the references to evaluate the fusion performance (see the red rectangles). Based on the eight fused images, the information of the corresponding regions is not fully described by ASR, GFF and GTTV. What is worse, in the right red rectangles, the artifacts can be observed in the fused images based on CSR, NSRCxWT, CBF and CNN. In comparison with other seven methods, the two regions in the fused image based on the proposed method are much better described.

Of course, there may be individual divergences during the evaluating process. To this end, the four metrics mentioned in subsection *B* are used to evaluate the fusion effects from more balanced and objective perspectives, and the numerical results are reported in Table 1, in which the value shown in bold in each row indicate the best result among the eight methods. Obviously, as for the first three metrics *Q*_*SF*_, *Q*_*Piella*_ and *Q*_*MI*_, the proposed method is almost always ranked the first. Owing to the special mechanism of GTTV, its *Q*_*CV*_ value is abnormal.

**Table 1:** Objective evaluation on the fused images based on different methods.

		**ASR**	**CSR**	**NSRCxWT**	**GFF**	**CBF**	**CNN**	**GTTV**	**Proposed**
Pair I	*Q_*SF*_*	34.8118	44.1029	42.4388	35.9596	36.8943	36.2532	34.5648	**45.2897**
	*Q_*Piella*_*	0.7094	0.7219	0.7299	0.7224	0.7302	0.7001	0.5910	**0.7520**
	*Q_*MI*_*	0.7083	0.8813	1.1378	0.6984	0.7198	0.7799	0.6727	**1.1507**
	*Q_*CV*_*	400.0300	367.5945	375.6842	402.3830	414.0351	302.5264	**830.0512**	423.3613
Pair II	*Q_*SF*_*	40.8550	**50.0756**	49.7400	39.9966	47.7477	44.3366	32.0796	49.9253
	*Q_*Piella*_*	0.7373	0.6991	0.7465	0.6587	0.7377	0.7431	0.5075	**0.7783**
	*Q_*MI*_*	0.6974	0.8798	1.0025	0.6704	0.7735	0.9054	0.6418	**1.0780**
	*Q_*CV*_*	1,145.383	1,290.245	716.1920	2,142.597	2237.970	971.9320	**3,762.081**	2535.860
Pair III	*Q_*SF*_*	39.0054	41.8544	40.3306	38.8861	38.1430	40.5021	27.7984	**42.5274**
	*Q_*Piella*_*	0.8974	0.9014	0.9009	0.9053	0.9012	0.8998	0.6221	**0.9193**
	*Q_*MI*_*	0.9498	1.0634	0.9922	0.9013	0.8901	0.9977	0.8141	**1.0675**
	*Q_*CV*_*	169.2490	161.1503	179.7873	150.0123	187.5749	139.1230	**1575.770**	177.0231
Pair IV	*Q_*SF*_*	28.4958	35.3432	36.7455	28.4490	32.4930	28.5946	24.0985	**36.9254**
	*Q_*Piella*_*	0.7667	0.8350	0.8295	**0.8408**	0.8612	0.7688	0.6847	0.8407
	*Q_*MI*_*	0.5002	0.7131	1.0356	0.5855	0.8597	0.5167	0.4761	**1.0553**
	*Q_*CV*_*	1,449.801	2,126.931	2,525.826	1,436.559	2,481.416	1,187.209	1,486.342	**2638.738**
Pair V	*Q_*SF*_*	28.1230	31.2896	31.5508	31.0568	31.5580	31.5670	11.9156	**32.4373**
	*Q_*Piella*_*	0.8102	0.9198	0.9118	0.9246	0.9213	0.9109	0.3985	**0.9265**
	*Q_*MI*_*	0.5846	0.9318	**1.0688**	0.8039	0.9030	1.0590	0.5759	1.0548
	*Q_*CV*_*	228.7973	18.3058	16.3897	25.1995	46.7478	16.3897	**985.4931**	231.8284
Pair VI	*Q_*SF*_*	27.2272	30.8178	31.3711	30.6399	30.6681	30.6681	11.9758	**31.6360**
	*Q_*Piella*_*	0.8237	0.9154	0.9133	0.9226	0.9189	0.9189	0.3655	**0.9249**
	*Q_*MI*_*	0.5806	0.9026	**1.0588**	0.7958	0.8377	0.8377	0.4874	1.0177
	*Q_*CV*_*	106.9227	8.0261	7.7041	28.8184	37.1281	7.7035	**822.7828**	127.5496

The bold values indicate the optimal results.

### Applications of the proposed method in other types of source images

**Figure 9:**
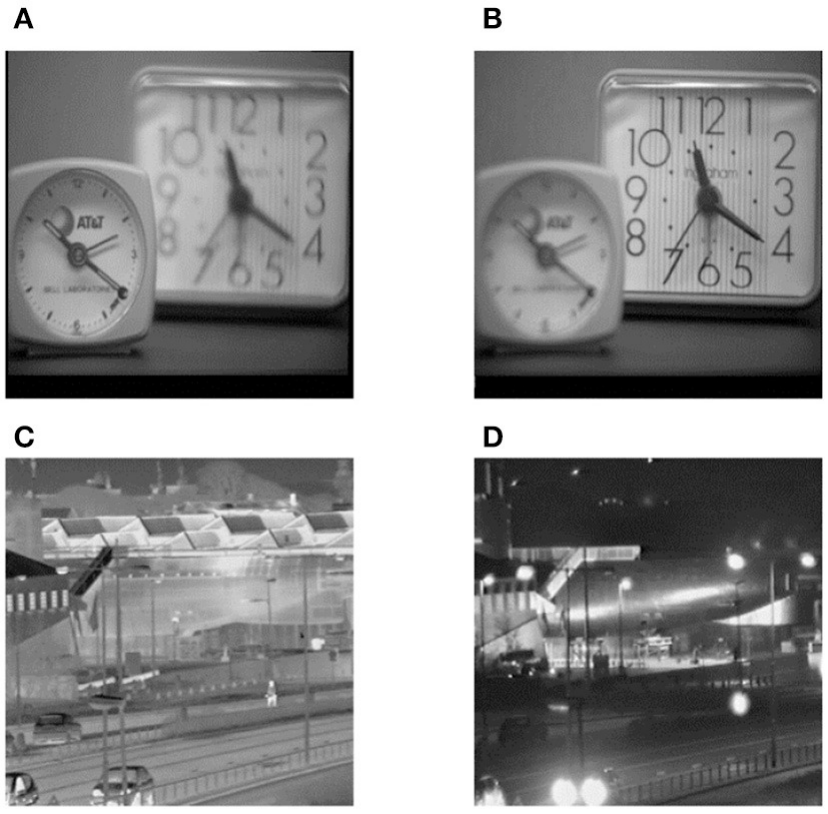
Another two types of source images. **(A)** Left-focus source image, **(B)** Right-focus source image, **(C)** Infrared source image, **(D)** Visible light source image.

Different types of images often have diverse characteristics. In order to verify and evaluate the comprehensive performance of the proposed method, extensive investigations on its usage in other types of source images are conducted in this subsection. Here, another two types of source images are selected, namely a pair of multi-focus source images and a pair of visible and infrared source ones, which are denoted by Pair VII and Pair VIII, respectively. These two pairs of source images are shown in Figure 9.

**Figure 10:**
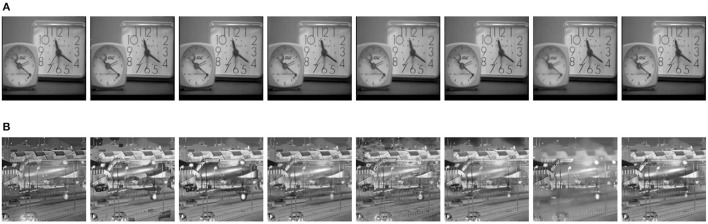
Fusion results on two pairs of source images with eight different methods. **(A)** Fusion results on Pair VII (from left to right: ASR, CSR, NSRCxWT, GFF, CBF, CNN, GTTV, Proposed). **(B)** Fusion results on Pair VIII (from left to right: ASR, CSR, NSRCxWT, GFF, CBF, CNN, GTTV, Proposed).

Apart from multimodal medical images, multi-focus images, gray and infrared images are also research hotspots in the field of image fusion. Therefore, these typical types of images are selected as the source images, and the corresponding fusion results are shown in Figure 10. In addition, the objective evaluation results are reported in Table 2. As can be observed, the fused images based on the proposed method are of satisfactory quality.

**Table 2:** Objective evaluation on the fused images based on different methods.

		**ASR**	**CSR**	**NSRCxWT**	**GFF**	**CBF**	**CNN**	**GTTV**	**Proposed**
Pair VII	*Q_*SF*_*	23.9632	24.1251	24.1067	24.4411	23.1825	24.3852	22.2385	**24.8856**
	*Q_*Piella*_*	0.9377	0.9328	0.9311	0.9325	0.9388	0.9323	0.9060	**0.9421**
	*Q_*MI*_*	1.0345	1.0905	1.1473	1.1031	1.0791	1.2059	1.1002	**1.2634**
	*Q_*CV*_*	54.6205	63.0834	64.7638	64.8442	64.2539	64.6459	**93.4914**	63.2364
Pair VIII	*Q_*SF*_*	30.2317	35.6011	35.5563	30.5951	33.6958	30.1220	22.2862	**35.9478**
	*Q_*Piella*_*	0.8227	0.8178	0.8045	0.8270	0.8341	0.7967	0.5837	**0.8345**
	*Q_*MI*_*	0.3698	0.6208	0.6356	0.3808	0.3833	**0.6467**	0.3255	0.6033
	*Q_*CV*_*	837.8217	1,298.0269	1,317.6476	1,209.4535	1,101.0403	1,325.8068	1,245.9047	**1,390.4678**

The bold values indicate the optimal results.

### Average running time of the proposed method

Typically, the visual effect as well as the metric values seems to be the focus of our attention. However, in the practical situations, the computational cost especially the average running time is also a very important factor we are interested in. In this subsection, the experimental results on Pair I are taken into consideration.

The hardware platform concerning the experiments above is as follows. A computer is equipped with an IntelCore i7-7700 3.60 GHz CPU and 16 GB memory. Besides, a GPU module GTX1060 is also employed here. All the simulation experiments are performed with matlab 2014b. In order to guarantee the objectivity of the experimental results, the same experiments are performed thrice *via* the proposed method, and then the average running time is calculated to be the final result. The statistics show that it only takes 1.32 s to achieve the final fused image *via* the proposed method, which is perfectly acceptable to the applications of the lesion detection and localization.

### Discussions on the potential research directions of the proposed method

Although the proposed method is proved to be effective to deal with the fusion issue of the multimodal medical images, it doesn't mean that there is no room for development of CNN theory. On the contrary, lots of researches and investigations are still required to be done in the future. To the best of our knowledge, the following several points are worth researching.

- Optimization of CNN architecture. It is well known that the birth of CNN is of epoch-making significance of the milestone for the area of image processing. However, the traditional CNN architecture has its own inherent drawbacks, which has been mentioned in Related work section. Therefore, the further researches on the optimization of CNN architecture are very necessary. On the one hand, CNN is a representative model in the deep learning field. The relation between the network depth of CNN and the final performance is always an interesting and meaningful topic. On the other hand, in this paper, the introduction of another theory is proved to be effective to overcome the above drawbacks of CNN to a certain extent, so the combination between CNN and other theories could be the future direction of development.
- As other typical fusion methods, the main structure is commonly composed of fusion models and fusion schemes. These two parts both play an instructive role in the whole process of image fusion. As for the fusion models, it has been involved in (a). Similarly, the investigations on the fusion schemes should also be emphasized in the future.

### Limitations of the proposed method

Despite its effectiveness, the proposed method also has its inherent limitations as follows.

Firstly, due to the nature of deep learning, the size of the training datasets determines the performance of the proposed method to a large extent. However, compared with the current well-known image datasets, the size of the medical image datasets suitable for training is usually small, so that the learning ability of the proposed network is limited. To solve this problem, the deep cooperation with domestic and foreign well-known medical institutions is necessary, and the construction of large medical image database is expectable.

Secondly, as the important component, ELM can significantly improve the execution efficiency of the proposed method, but its nonlinear representation ability is not well. Therefore, how to improve the classical ELM to optimize the representation ability of nonlinear features becomes a research direction in the future.

## Conclusions

In this paper, a novel fusion method called CELM is proposed to deal with the fusion issue of multimodal medical images. CELM combines the advantages of both CNN and ELM. Compared with other typical fusion methods, the proposed one has obvious superiorities in terms of both subjective visual quality and objective metric values. In addition, the potential research directions of the proposed method are also given and discussed, the contents of which will be the emphasis of our next work in future.

## Data availability statement

The data generated during the current study are not publicly available due to funding restrictions.

## Author contributions

Conceptualization: WK and CL. Methodology: WK. Software and validation: CL. Writing: CL and YL. All authors contributed to the article and approved the submitted version.

## Funding

This research was partially supported by the National Natural Science Foundation of China (Grant Number 61902296) and the Natural Science Foundation of Shaanxi Province of China (Grant Number 2022JM-369).

## Conflict of interest

The authors declare that the research was conducted in the absence of any commercial or financial relationships that could be construed as a potential conflict of interest.

## Publisher's note

All claims expressed in this article are solely those of the authors and do not necessarily represent those of their affiliated organizations, or those of the publisher, the editors and the reviewers. Any product that may be evaluated in this article, or claim that may be made by its manufacturer, is not guaranteed or endorsed by the publisher.

## Footnotes

^1^ http://www.med.harvard.edu/AANLIB/home.htm

^2^ http://www.imagefusion.org
